# The effectiveness of the semi-virtual simulation teaching model based on the Standards of Best Practice of the International Nursing Association for Clinical Simulation and Learning

**DOI:** 10.1016/j.ijnss.2025.12.002

**Published:** 2025-12-12

**Authors:** Peizhuo Shi, Ping Yang, Jingzhi Zhuang, Yanru Wang, Dong Pang, Qian Lu, Sanli Jin, Jinxiao Zhao, Wei Chen, Ke Li, Xiangping Li

**Affiliations:** a School of Nursing, Baotou Medical College, Baotou, China; b School of Nursing, Peking University, Beijing, China; c College of Arts and Sciences, Cornell University, New York, USA; d Department of Outpatient, Peking University People’s Hospital, Beijing, China; e Department of Ophthalmology, Peking University Third Hospital, Beijing, China; f Office of Education, Peking University First Hospital, Beijing, China

**Keywords:** Nursing education, Nursing students, Quasi-experimental study, Semi-virtual simulation, Standards of Best Practice

## Abstract

**Objectives:**

This study aimed to compare the effectiveness of the semi-virtual simulation and traditional simulation teaching models based on the Standards of Best Practice (SOBP) according to the International Nursing Association for Clinical Simulation and Learning (INACSL) in the Adult Nursing course.

**Methods:**

This study used a quasi-experimental design. A total of 94 third-year nursing students from a university in Beijing between November and December 2022 were recruited as participants. An innovative semi-virtual simulation teaching model was designed based on the SOBP established by the INACSL. In the Adult Nursing course, both the semi-virtual and traditional simulation teaching models were implemented. At the end of the simulation sessions, participants completed the Chinese version of the Simulation Effectiveness Tool–Modified (SET-M) to assess the effectiveness of the two teaching models.

**Results:**

All nursing students completed the simulation sessions. There was no difference (*t* = −0.93, *P* = 0.353) in the total scores between the semi-virtual simulation teaching model (50.87 ± 5.30) and the traditional simulation teaching model (50.37 ± 5.16). However, there was a statistically significant difference (*t* = −2.65, *P* = 0.010) in the prebriefing section (semi-virtual simulation: 5.60 ± 0.71; traditional simulation: 5.33 ± 0.78). In contrast, no statistically significant differences were found for the scenario and debriefing sections (*P* > 0.05). At the individual item level, statistical differences (*P* < 0.05) between the two models were identified for items 1 and 9, but not for the remaining items (*P* > 0.05). By analyzing the open-ended question, it was found that both simulation models were effective, and students’ comments were similar.

**Conclusions:**

The study demonstrated equivalent effectiveness between the semi-virtual and traditional simulation teaching models. Semi-virtual simulation teaching model could offer a more flexible and feasible approach to simulation teaching.

## What is known?

• Virtual simulation technologies, including virtual reality and augmented reality, have been shown to significantly improve nursing students’ problem-solving skills, communication skills, and core professional competencies with small to large effect sizes, supporting their role as effective tools in clinical nursing education.
• High-quality simulation in nursing education is designed based on the Standards of Best Practice (SOBP) according to the International Nursing Association for Clinical Simulation and Learning (INACSL). Although the efficacy of virtual and traditional simulation methods has been demonstrated, few studies have evaluated the effectiveness of the semi-virtual simulation teaching model aligned with the SOBP.

## What is new?

• This study demonstrated that a designed semi-virtual simulation teaching model was as effective as the traditional simulation teaching model when applied in the Adult Nursing course.
• Notably, semi-virtual simulation teaching model showed statistically significant advantages in the prebriefing section, highlighting its effectiveness in enhancing the preparation phase of clinical learning for nursing students.
• Semi-virtual simulation teaching model may provide a more flexible and feasible way for nursing faculty to learn about simulation teaching methods and immerse themselves in a standardized simulation process.

## 1 Introduction

Simulation is indispensable in medical training, particularly for disciplines such as nursing, which rely heavily on practical experience [1]. Core nursing courses require a broad knowledge base and strong practical skills, and mastery of these core courses is crucial for cultivating competent professionals, particularly in the context of global aging. Widely adopted in nursing education globally, simulation replicates clinical scenarios to maximize the acquisition of skills and knowledge [1,2]. It has been applied in courses such as critical care, pediatric nursing, obstetric nursing, and health assessment [3]. Simulation enables immersive practice, learning, and evaluation by creating realistic, risk-free, and repeatable high-fidelity clinical environments. Repeated practice stimulates hippocampal and prefrontal theta-band mechanisms to form implicit memory [4], and this implicit memory further provides effective contextual cues for subsequent clinical practice. Facilitators can also conduct comprehensive debriefings and guided feedback [1], enhancing students’ clinical decision-making, problem-solving, critical thinking, confidence, and psychological resilience [5,6].

Digital transformation is reshaping nursing education, with virtual simulation emerging as a pivotal pedagogical tool. Virtual simulation, which encompasses technologies such as virtual and augmented reality, creates immersive and risk-free learning environments for clinical purposes [7]. Recent systematic evidence [8] has demonstrated that virtual simulation technologies, including virtual and augmented reality, have a significantly positive impact on nursing education outcomes. Specifically, these technologies improve problem-solving skills (effect size, 0.2–0.9), communication skills (effect size, 0.4–0.7), and core professional competencies (effect size, 0.3–0.9), with small to large effect sizes, supporting their potential as practical tools in clinical nursing education [8]. Digital education offers advantages such as reduced costs, enhanced equity, and a narrowing of educational disparities [9,10]. It also boosts student engagement, cultivates digital literacy [11,12], and achieves comparable or superior outcomes to traditional methods [8].

Building upon these advancements in digital education, a spectrum of simulation modalities has evolved. Among these, the integration of digital and physical learning spaces represents a particularly promising frontier, as it addresses critical limitations inherent to single-modal approaches. During the COVID-19 pandemic, routine healthcare and education systems were disrupted, posing immense challenges to higher education [13]. According to statistics from the United Nations Educational, Scientific and Cultural Organization, as of April 2020, over 1.6 billion students had been affected, prompting worldwide innovations in large-scale teaching models [14]. This accelerated the shift from in-person to online or hybrid instruction, effectively maintaining educational continuity and outcomes despite physical distancing measures [15]. Meanwhile, amid the digitalization trend in education, evolving technologies, societal changes, and growing learner demands, adapting teaching strategies and diversifying pedagogical approaches have become necessary [16,17]. Current methods include synchronous/asynchronous online teaching, as well as blended online-offline models, which meet the demands of the digital education revolution [18]. The integration of digital and physical learning spaces is a key frontier, as it addresses critical limitations of existing single-modal approaches.

Given the concurrent advancements in connectivity, digital pedagogy, globalization, and AI, there is a pressing need to innovate simulation formats that leverage the benefits of remote instruction without sacrificing the fidelity of physical practice. High-quality simulation in nursing education is defined by adherence to the Standards of Best Practice (SOBP) of the International Nursing Association for Clinical Simulation and Learning (INACSL) [19,20]. We propose an online instructor-guided semi-virtual simulation teaching model, where teachers remotely guide the session, regulate pacing, and provide structured debriefing. At the same time, students conduct hands-on simulations in physical skills laboratories. This approach aimed to provide environmental familiarity, technical practice, and immersive scenarios while enabling facilitators to deliver simulation teaching via the cloud. A key innovation of the proposed model is the synchronous combination of hands-on student practice in the physical lab and real-time, remote guidance from instructors. This study aimed to validate the effectiveness of the semi-virtual simulation teaching model through a quasi-experimental research design.

## 2 Methods

### 2.1 Study design and participants

In accordance with the Transparent Reporting of Evaluations with Nonrandomized Designs (TREND) statement [21], a quasi-experimental design was employed. All third-year undergraduate nursing students using cluster sampling at a university in Beijing between November and December 2022 were recruited as participants. These students had completed basic learning of theory and skills, including foundational and clinical nursing courses. Inclusion criteria were as follows: 1) aged 18 years or older; 2) undergraduate nursing students; 3) participated in the Adult Nursing course. Exclusion criteria included: 1) those unable to participate fully in two scenario-based simulations; 2) presence of cognitive disorders or psychiatric conditions.

### 2.2 Sample size

To compare the effectiveness of two different simulation teaching methods, with the expectation that semi-virtual simulation teaching will achieve results similar to those of traditional simulation teaching, a non-inferiority test was used to calculate the sample size. Based on the effective scores (50.27 ± 5.30), students evaluated using the Chinese version of the Simulation Effectiveness Tool-Modified (SET-M) after we used the traditional simulation teaching model for the duplicate course content last year. The sample size was calculated using the following [22]: *n* = (*Z*_1-*α*_+*Z*_1-*β*_)^2^ × *σ*_α_^2^/(*δ*-|*μ*_1_-*μ*_2_|)^2^. With *α* = 0.05 (two-tailed), *β* = 0.20, *Z*_1-*α*_ = 1.96, *Z*_1-*β*_ = 0.84, *σ*_a_ = *σ* = 5.3, *δ* = 2 (in non-inferiority testing, generally *δ* = 2) and expected true difference |*μ*_1_-*μ*_2_| ≈ 0. The calculated minimum sample size was 55. A total of 94 third-year nursing students were enrolled, which exceeded the sample size requirement.

### 2.3 Traditional simulation teaching

Traditional simulation teaching creates highly realistic clinical scenarios, allowing learners to engage in immersive training within a risk-free environment. Instructors provide guided feedback to help students enhance their clinical decision-making, problem-solving, and critical thinking skills. This approach has been integrated into the Adult Nursing course at our Nursing School for three years.

#### 2.3.1 Instructors and students

##### 2.3.1.1 Simulation teaching team

The core simulation teaching team for this course comprised seven members who served as debriefing facilitators. All seven faculty members (professors, associate professors, or lecturers) had received simulation teaching training, with one additionally certified as a National League for Nursing (NLN)-authorized trainer for simulation instructors. In addition to the core teaching team, other personnel played crucial supporting roles in the simulation sessions. This included clinical nurse specialists (who actively participated in scenario running by role-playing as physicians or patients’ family members to facilitate scenario progression and enhance clinical realism), simulation lab assistants (who provided essential technical support for SimMan 3G system or manikin operation, equipment configuration, and environmental setup to ensure scenario fidelity), and teaching assistants (who assisted with course coordination and operational logistics, including material preparation, time management, and participant guidance during simulation implementation).

##### 2.3.1.2 Instructor composition

Each simulation was facilitated by a dedicated team of at least four instructors. This team consisted of a faculty member, a clinical nurse specialist from teaching hospitals, a laboratory assistant, and a teaching assistant.

##### 2.3.1.3 Students

Nursing students were grouped based on their class divisions (a total of three classes, each class was divided into two groups), with each group comprising 15–16 students, resulting in a total of six groups. Two students from each group, selected by random drawing, participated in the simulation exercises, where they played the roles of a primary nurse and an assisting nurse, respectively. The remaining 13–14 students served as observers during the scenario, completing observation forms.

#### 2.3.2 Three stages of traditional simulation teaching

##### 2.3.2.1 Prebriefing

One week prior to the simulation, preparation requirements were released for students, along with extended learning resources (e.g., best practice guidelines, relevant literature). The laboratory was open for students to practice (knowledge/skills). Teachers met to prepare standardized teaching, including simulation cases, guided content, objectives, and Standardized Patient (SP) training. On the day of the simulation, the facilitator completed the briefing; simulation lab assistants tested and ran the SimMan 3G system.

##### 2.3.2.2 Scenario

The student participants implement nursing care for SimMan 3G in accordance with simulation objectives. Clinical nurse specialists played the role of doctors/family members and helped students achieve simulation goals; the simulation lab assistants ensured that the system operated according to the case design content. Student observers observed through one-way mirrors and filled out observation forms.

##### 2.3.2.3 Debriefing

All students and facilitators participanted in face-to-face feedback. Facilitators guide reflective summarization (assessment, decision-making, communication, safety, teamwork). Debriefing covers personal gains, skill performance, and team outcomes. Facilitators evaluate students’ attitudes, expressions/behaviors, as well as their verbal communication.

### 2.4 Semi-virtual simulation teaching

#### 2.4.1 Characteristics of the semi-virtual simulation teaching

In addition to containing duplicate teaching content, semi-virtual simulation teaching model has the following key features.

##### 2.4.1.1 Online and offline synchronous collaboration

Using the Tencent meeting tool and WeChat group chats, a synchronous collaborative network spanning multiple locations was established. This enables instructors to provide remote teaching guidance and real-time interaction with students, demonstrating the application of integrated online and offline synchronous simulation in nursing education.

##### 2.4.1.2 Multimodal learning environment

Through on-site multi-angle observation cameras and real-time audio-visual monitoring, the teaching environment is dynamically optimized, highlighting the advantages of “multimodal learning” and accommodating the diverse learners’ perceptual preferences.

##### 2.4.1.3 Real-time interaction remotely

With the help of simulation lab assistants, teachers can remotely monitor students’ performance in simulation teaching in real-time, while students can receive timely guidance and feedback from teachers, ensuring the effective operation of simulation teaching.

#### 2.4.2 Implementation of semi-virtual simulation teaching

All students who participated in traditional simulation learning also participated in semi-virtual simulation learning.

##### 2.4.2.1 Prebriefing

During the preparation phase, the simulation instructor team verified network reliability, software functionality, and system stability one day before the simulation session. The facilitators then conducted the briefing phase online.

##### 2.4.2.2 Scenario

Participants reported patient conditions online; clinical nurse specialists remotely played the role of doctors, providing real-time facilitation based on simulation objectives; simulation lab assistants were responsible for monitoring audio and video quality, coordinating multiple cameras, and ensuring network stability; a WeChat group was used for timely communication among teaching team members.

##### 2.4.2.3 Debriefing

All students and facilitators joined an online meeting room for remote face-to-face feedback. Tencent meeting app enabled real-time data acquisition.

### 2.5 Measures

#### 2.5.1 Sociodemographic data

Sociodemographic data include sex, age, and grade of the nursing students.

#### 2.5.2 Evaluation of the effectiveness of simulation teaching

The Chinese version of the SET-M [23] was utilized to assess students’ perceptions of the effectiveness of simulation-based learning. Leighton et al. [24] developed the SET-M by adapting the original simulation effectiveness tool to align with INACSL’s best practice standards. It provides a structural evaluation of the simulation process, including prebriefing, scenario implementation, and debriefing, enabling comprehensive quality assessment of simulation education [23]. The SET-M consists of 20 items-19 closed-ended and one open-ended question. It is divided into four dimensions: prebriefing (items1-2), learning (items 3–8), confidence (items 9–14), and debriefing (items 15–19). A 3-point Likert scale was used (1 = “strongly disagree” to 3 = “strongly agree”), and the aggregate score range was 19–57, with higher scores indicating better simulation design and implementation, as well as greater student satisfaction. The Chinese version was translated by Li et al. [23] and has been extensively applied in relevant Chinese studies [[25], [26], [27], [28]]. The Chinese version of the SET-M maintains the original structure and the total Cronbach’s *α* coefficient is 0.89. To better understand students’ learning experiences, qualitative data were collected through item 20’s open-ended questionn (What else would you like to say about today’s simulated clinical experience?), inviting students to share comments and suggestions about the simulation.

### 2.6 Data collection

Students’ general information and the Chinese version of the SET-M were collected on-site using a paper-based questionnaire. Before simulation teaching, students’ demographic data were collected, and scores were obtained twice: after traditional simulation teaching and after semi-virtual simulation teaching. After each simulation session, teaching assistants distributed questionnaires to all students on-site and explained how to complete them. The questionnaires were anonymous, and students were asked to answer the open-ended question voluntarily. After the questionnaires were completed, the teaching assistants checked and collected them. All 94 students completed and submitted the questionnaires after two simulation sessions, with a 100 % valid response rate.

### 2.7 Data analysis

Data were entered into an Excel spreadsheet and analyzed using SPSS 26.0. Continuous variables were summarized using means and standard deviations (*SD*), while categorical variables were described using frequencies and percentages. For continuous data, the Kolmogorov-Smirnov test was used to test the normality of the data. Then, normally distributed variables were analyzed using paired samples *t*-tests, whereas non-normally distributed variables were assessed using Rank-Sum test. A significance level of *P* < 0.05 was used to determine statistical significance. Content analysis was used to analyze the results of this open-ended question.

### 2.8 Ethical considerations

This study was conducted in accordance with the principles outlined in the Declaration of Helsinki and China’s Measures for the Ethical Review of Science and Technology. The research protocol has been reviewed and approved by the Ethics Committee of the School of Medicine, Peking University (IRB00001052-19031). All students who participated in the study were fully informed and provided with a consent form.

## 3 Results

### 3.1 Characteristics of the participants

During the study, 94 third-year undergraduate nursing students attended simulation teaching. There were 36 males (38.3 %) and 58 females (61.7 %), with an average age of 20.49 ± 0.88 years.

### 3.2 Comparison of effectiveness scores between two teaching models

The paired samples *t*-test results showed no statistically significant difference in total scores between the two teaching models. Specifically, the traditional simulation teaching model had an average score of 50.37 ± 5.16, while the semi-virtual simulation teaching model averaged 50.87 ± 5.30. Although the semi-virtual simulation teaching model’s mean score was slightly higher by 0.5 points, the statistical test results (*t* = −0.93, *P* = 0.353) indicated that this difference was not statistically significant, and no significant difference was observed between the two models in terms of total scores.

For the prebriefing section, the mean score for the semi-virtual simulation teaching model was higher than the traditional simulation model (5.60 ± 0.71 vs. 5.33 ± 0.78; *t* = *-*2.65; *P* = 0.010). For the two dimensions in the scenario phase (learning and confidence dimensions) and the debriefing phase (debriefing dimension), there is no statistical difference between the two models (*P* > 0.05). The mean scores for items 1 and 9 were higher in the semi-virtual simulation teaching model than in the traditional simulation teaching model (*t* = −3.57 and −2.47, respectively; *P* < 0.05). The scores for the other 17 items did not differ between the two models (*P* > 0.05).The detailed score comparisons for the tool were shown in Table 1.

**Table 1:** Comparison of scores on the Chinese version of the Simulation Effectiveness Tool - Modified between traditional simulation and semi-virtual simulation teaching models (*n* = 94).

Dimensions and items	Traditional simulation	Semi-virtual simulation	*t*	*P*
Prebriefing	5.33 ± 0.78	5.60 ± 0.71	−2.65	0.010
1. Prebriefing increased my confidence	2.52 ± 0.52	2.74 ± 0.44	−3.57	0.010
2. Prebriefing was beneficial to my learning	2.81 ± 0.40	2.85 ± 0.39	−0.78	0.436
Learning	15.61 ± 1.89	15.67 ± 2.09	−0.24	0.815
3. I am better prepared to respond to changes in my patient’s condition	2.60 ± 0.51	2.69 ± 0.47	−1.30	0.196
4. I developed a better understanding of the pathophysiology	2.55 ± 0.52	2.51 ± 0.54	0.63	0.530
5. I am more confident of my assessment skills	2.50 ± 0.54	2.55 ± 0.52	−0.76	0.449
6. I felt empowered to make clinical decisions	2.63 ± 0.53	2.60 ± 0.52	0.47	0.642
7. I developed a better understanding of medications. (Leave blank if no medications in scenario)	2.68 ± 0.47	2.61 ± 0.51	1.04	0.299
8. I had the opportunity to practice my clinical decision-making skills	2.66 ± 0.48	2.71 ± 0.48	−0.84	0.401
Confidence	15.14 ± 2.50	15.55 ± 2.24	−1.72	0.089
9. I am more confident in my ability to prioritize care and interventions	2.53 ± 0.56	2.69 ± 0.49	−2.47	0.015
10. I am more confident in communicating with my patient	2.55 ± 0.54	2.63 ± 0.53	−1.22	0.225
11. I am more confident in my ability to teach patients about their illness and interventions	2.51 ± 0.54	2.52 ± 0.54	−0.16	0.877
12. I am more confident in my ability to report information to health care team	2.53 ± 0.54	2.61 ± 0.51	−1.19	0.239
13. I am more confident in providing interventions that foster patient safety	2.62 ± 0.51	2.68 ± 0.49	−1.03	0.306
14. I am more confident in using evidence-based practice to provide care	2.39 ± 0.57	2.43 ± 0.58	−0.48	0.633
Debriefing	14.31 ± 1.21	14.05 ± 1.46	1.68	0.353
15. Debriefing contributed to my learning	2.90 ± 0.30	2.87 ± 0.34	0.83	0.408
16. Debriefing allowed me to communicate my feelings before focusing on the scenario	2.82 ± 0.39	2.76 ± 0.46	1.14	0.259
17. Debriefing was valuable in helping me improve my clinical judgment	2.87 ± 0.34	2.80 ± 0.43	1.47	0.145
18. Debriefing provided opportunities to self-reflect on my performance during simulation	2.88 ± 0.32	2.85 ± 0.36	0.73	0.470
19. Debriefing was a constructive evaluation of the simulation	2.84 ± 0.37	2.78 ± 0.41	1.15	0.254
Total score	50.37 ± 5.16	50.87 ± 5.30	−0.93	0.353

*Note:* Data are *Mean* ± *SD*.

### 3.3 The experiences of semi-virtual simulation teaching

After the semi-virtual simulation teaching, a total of 17 students answered the open-ended question. Students generally felt that semi-virtual simulation teaching was as effective as traditional simulation teaching. For instance, one student responded, *“Even though the teachers were online (as opposed to in-person in traditional simulation teaching), I also gained a great deal through the semi-virtual simulation teaching").* The feedback was categorized into three main categories. 1) Semi-virtual simulation teaching demonstrated comparable effectiveness to traditional simulation teaching in promoting knowledge comprehension and application. (sample response: *“Semi-virtual simulation teaching of clinical scenarios provided effective practical application of theoretical knowledge, with clear course objectives and significant learning outcomes”).* To summarize, semi-virtual simulation teaching is conducive to the understanding of knowledge. 2) Semi-virtual simulation teaching model is conducive to combining theory and practice (sample response: *“Sometimes, we have mastered the knowledge, yet without experience of its application, we could panic in settings of practice. Semi-virtual simulation teaching helps us develop proficiency in utilizing our knowledge”*). 3) Semi-virtual simulation teaching model is conducive to improving skills and mental readiness (sample response: *“Participating in role play, I am much more confident this time. This is probably attributable to my grasp of knowledge and the Visual Interactive Simulation—these are very useful formats. We learn clinical knowledge from it”).*

## 4 Discussion

### 4.1 Semi-virtual simulation teaching demonstrates teaching effectiveness comparable with that of traditional simulation teaching

The results of this study indicate that the efficacy of the semi-virtual simulation model is no different from that of the traditional model. Compared with the traditional simulation model, the SET-M’ (Chinese version) total score and the scores for the second and third dimensions showed no statistically significant differences (*P* > 0.05). Babaita et al. [29] compared 360° VR learning and traditional face-to-face simulation teaching in a closed tracheal suction experiment, showing that the digitally based teaching method is as effective as traditional teaching. The study confirmed the effectiveness of VR technology in helping students practice procedures. In contrast, our study demonstrated that even in more complex simulation cases, combining real-time audio-visual technology can achieve the same results as traditional simulation teaching. Omole et al. [30] compared the effects of online and in-person teaching methods for medical students in their first and second years over four semesters. The comparison between online and in-person teaching, conducted over two semesters each for the intervention group, showed no statistically significant differences in student performance. This study validated that remote practical teaching is as effective as offline teaching, and our research further validated the effectiveness of remote simulation teaching. This may be attributed to the fact that implementing remote simulation teaching for practical courses in medical education ensures a high level of students’ perception of their educational environment, thereby promoting students’ learning commitment [31]. Additionally, the content analysis of students’ open-ended question reveals that they believe the semi-virtual simulation teaching model can achieve the same learning outcomes as traditional simulation teaching. Under the premise of similar overall teaching effectiveness, the semi-virtual simulation teaching model ensures training in skills and increases the flexibility of teaching locations for facilitators. With teachers conducting remote simulation teaching, this model can facilitate collaboration among institutions at different levels, thereby promoting the fluidity and fairness of educational resources. At the same time, the shortage of qualified domestic teachers for simulation teaching is concerning [32]. Few teachers in China have obtained accreditation from the NLN Accrediting Commission (NLNAC). In the future, it may be worthwhile to explore the possibility of collaboration in training and certification among NLNAC-accredited facilitators in the United States, accredited domestic facilitators, and other teachers.

### 4.2 The semi-virtual simulation teaching model can enhance students’ learning confidence

The results demonstrate a statistically significant difference in item 1 of the prebriefing section between teaching formats (*t* = *-*3.57*, P* < 0.05), indicating that students reported stronger agreement with “prebriefing increased my confidence” in semi-virtual simulation teaching compared to traditional simulation teaching. This advantage may be attributed to several factors inherent to the semi-virtual format. First, eliminating faculty commuting time and reducing environmental transitions allows facilitators to dedicate more effort to developing effective instructional content. Second, the format enables more targeted learning design and better attention to individual student needs during prebriefing, thereby enhancing the establishment of psychological safety [33,34]. These preparatory elements are fundamental to ensuring optimal teaching outcomes [35].

Furthermore, the remote teaching component of semi-virtual simulation reduces the explicit and implicit control that facilitators have over the learning process. Emerging evidence suggests that this may lead to physiological changes in students, including decreased cortisol levels and increased heart rate variability [36,37], which are associated with lower stress levels and, consequently, greater learning confidence [6,8]. We therefore recommend that facilitators implementing semi-virtual simulation devote particular attention to preparatory guidance to maximize its educational value.

The results also revealed significant higher scores for item 9 (“I am more confident in my ability to prioritize care and interventions”) in the semi-virtual simulation teaching model (*t* = −2.47, *P* < 0.05), This finding may be partially explained by the digital features of online teaching (e.g., audio/video recording and playback capabilities) that promote students’ engagement and preparation [38]. However, we must also consider the potential influence of case characteristics, as the traditional simulation’s acute myocardial infarction scenario (progressing to cardiac arrest) presented different priority recognition challenges compared to the more gradual progression of compartment syndrome in the semi-virtual simulation. In semi-virtual simulation teaching, facilitators control the simulation process online, while students reinforce their skills through in-person simulation, resulting in a parallel, same-pace learning process. In the era of digital teaching methods, this teaching model aligns with the practicality of nursing training and the current educational trend. It is expected to become a new teaching model in the background of digital teaching. Moreover, artificial intelligence technology can even be integrated to conduct exploratory analysis of the implementation effect of this teaching strategy [39,40].

## 5 Limitations

This study has several limitations inherent to its single-center design, which may constrain the generalizability of findings to other regions or cultures. Additionally, since the students participating in this study had previously engaged in traditional simulation teaching on multiple occasions, they were very familiar with the process of traditional simulation teaching. This familiarity may have some influence on the findings of this study. Additionally, slight variations in case difficulty between the two learning modalities may have introduced some bias. However, the transition from traditional to semi-virtual simulation teaching within the same cohort reflects authentic educational outcomes under the constraints of COVID-19, when in-person simulation was no longer feasible.

This study was conducted at our Nursing School, which may limit the generalizability of the findings. Participants’ prior extensive simulation experience (a characteristic of this institution) may limit applicability to simulation learning beginners. Future research should validate these findings through multicenter studies with larger, diversified samples using standardized cases, while implementing robust quality control measures for simulation delivery.

## 6 Conclusions

The semi-virtual simulation teaching model represents an innovative approach to teaching and learning. Overall, this approach proves to be as effective as traditional simulation teaching model. However, semi-virtual simulation is more effective in increasing students’ confidence during the prebriefing phase, identifying care to prioritize, and enhancing confidence in intervention skills. Moving forward, this model can be implemented in nursing schools that lack qualified simulation instructors to advance standardized simulation teaching practices and improve the quality of nursing practice education.

## CRediT authorship contribution statement

**Peizhuo Shi:** Conceptualization, Data curation, Formal analysis, Writing - original draft, Writing - review & editing. **Ping Yang:** Conceptualization, Methodology, Validation, Formal analysis, Writing - review & editing, Project administration, Resources. **Jingzhi Zhuang:** Formal analysis, Writing - original draft, Writing - review & editing. **Yanru Wang:** Formal analysis, Writing - review & editing. **Dong Pang:** Methodology, Investigation, Writing - review & editing. **Qian Lu:** Methodology, Investigation, Writing - review & editing. **Sanli Jin:** Methodology, Investigation, Writing - review & editing. **Jinxiao Zhao:** Investigation, Data curation, Writing - review & editing. **Wei Chen:** Investigation, Data curation, Writing - review & editing. **Ke Li:** Investigation, Data curation, Writing - review & editing. **Xiangping Li:** Conceptualization, Methodology, Validation, Formal analysis, Writing - review & editing, Project administration, Supervision, Resources.

## Data availability statement

The datasets generated during and/or analyzed during the current study are available from the corresponding author upon reasonable request.

## Funding

None.

## Declaration of competing interest

The authors have declared no conflict of interest.
