# Supplementary material for: The effectiveness of the semi-virtual simulation teaching model based on the Standards of Best Practice of the International Nursing Association for Clinical Simulation and Learning
Source: Int J Nurs Sci. 2025 Dec 12;13(1):61–7. doi: 10.1016/j.ijnss.2025.12.002 (PMC12891799; doi:10.1016/j.ijnss.2025.12.002)
Supplement: Multimedia component 1 [file mmc1.docx]

**基于最佳实践标准的半虚拟仿真教学模式的应用效果研究**

**史培卓，杨萍，庄敬之，王艳茹，庞冬，路潜，金三丽，赵金宵，陈巍，李珂，李湘萍**

【**摘要】**

**目的** 基于国际临床护理模拟教学学会（International Nursing Association for Clinical Simulation and Learning, INACSL）制定的模拟教学最佳实践标准（Standards of Best Practice, SOBP），比较《成人护理学》课程中半虚拟模拟与传统模拟教学模式的教学效果。

**方法** 采用类实验研究设计，于2022-2023学年第一学期，整群选取94例北京一所高校三年级护理本科生作为研究对象。依据INACSL制定的模拟教学SOBP，构建了一种创新的半虚拟模拟教学（线上与线下融合）模式。在《成人护理学》实践教学中，分别采用半虚拟模拟与传统模拟教学模式进行授课。课程结束后，使用中文改良版模拟效果评价工具（Simulation Effectiveness Tool-Modified , SET-M）对两种教学模式的教学效果进行评估。

**结果** 94例学生均完成了两种教学模式。结果显示，半虚拟模拟教学（50.87 ± 5.30）与传统模拟教学（50.37 ± 5.16）的SET-M总分差异比较无统计学意义（*t*=-0.93，*P*=0.353）。然而，在模拟前介绍维度，半虚拟模拟教学（5.60±0.71）得分高于传统模拟教学（5.33±0.78），差异有统计学意义（*t*=-2.65，*P*=0.010）；而在模拟案例运行维度与引导性反馈维度，两种模拟教学模式得分差异无统计学意义（*P*>0.05）。单个条目分析显示，仅条目1与条目9的得分差异具有统计学意义（*P*<0.05），其余条目均无明显差异（*P*>0.05）。对SET-M开放性问题的归纳分析表明，两种教学模式均被学生认为有效，且评价较为相似。

**结论** 半虚拟模拟教学模式与传统模拟教学模式效果相当，为模拟教学提供了一种更为灵活可行的实施路径。
【**关键词**】护理教育；护生；类实验研究；半虚拟教学；最佳实践标准

**通信作者：**李湘萍，E-mail：[xiangping@bjmu.edu.cn](mailto:xiangping@bjmu.edu.cn)
